# Supplementary material for: Leishmania Ribosomal Protein (RP) paralogous genes compensate each other’s expression maintaining protein native levels
Source: PLoS One. 2024 May 16;19(5):e0292152. doi: 10.1371/journal.pone.0292152 (PMC11098316; doi:10.1371/journal.pone.0292152)

**S1Fig.** **Strategy used for gene tagging and its confirmation for the ribosomal proteins under study.** (A) Diagram of the pPLOT plasmid from which a linear fragment was amplified by general PCR and used as donor-DNA is shown. The insertion of the tag at 5’-end of each gene copy was confirmed by PCR due to the amplicon length differences between parental cell line (wt) and tagged version (tag); in (B) the predicted length of the amplicon and in (C) electrophoretic fractionation of PCR products. (D) *in silico* model for the three-dimensional RPL13a paralogues showing the identical 3D structures for both proteins.


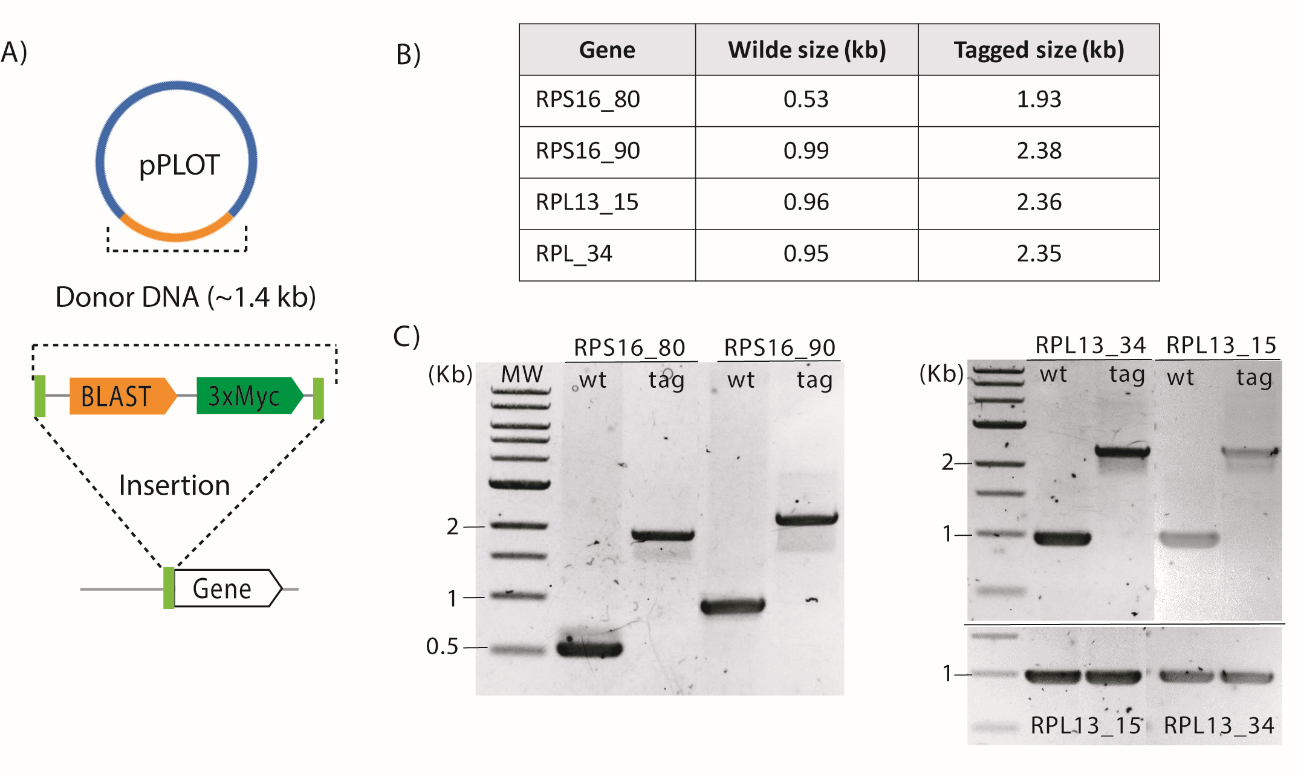


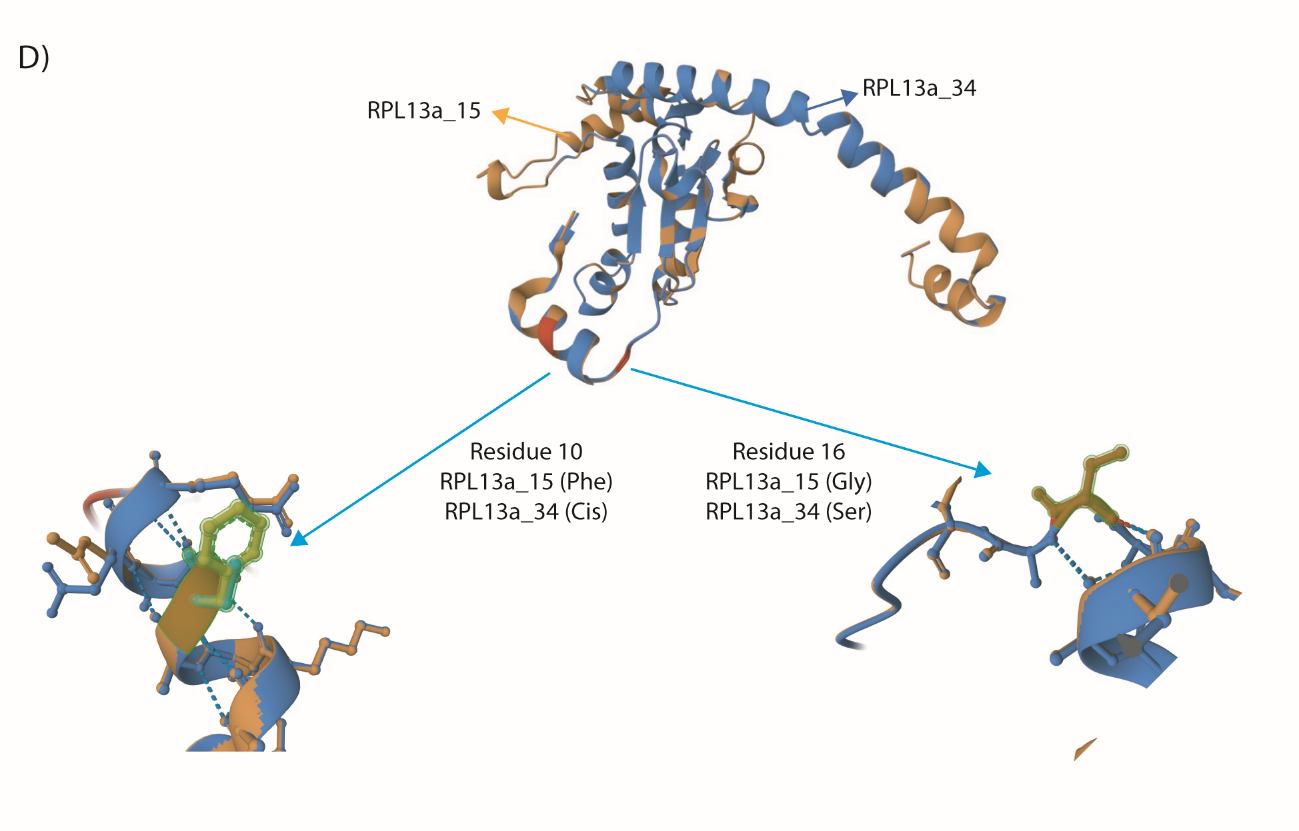

Supplement: S1 Fig — (DOCX) [file pone.0292152.s001.docx]
