# Supplementary material for: Leishmania Ribosomal Protein (RP) paralogous genes compensate each other’s expression maintaining protein native levels
Source: PLoS One. 2024 May 16;19(5):e0292152. doi: 10.1371/journal.pone.0292152 (PMC11098316; doi:10.1371/journal.pone.0292152)

**S2Fig.** **Immunodetection of RPS16 using a specific rabbit antibody**. α-RPS16 antibody was used for the identification of both RPS16 paralogues in procyclic and metacyclic promastigotes (in green). Nucleus (N) and kinetoplast (K) are shown in blue by Hoescht staining.

RPS16 protein detection by α-RPS16 antibody.


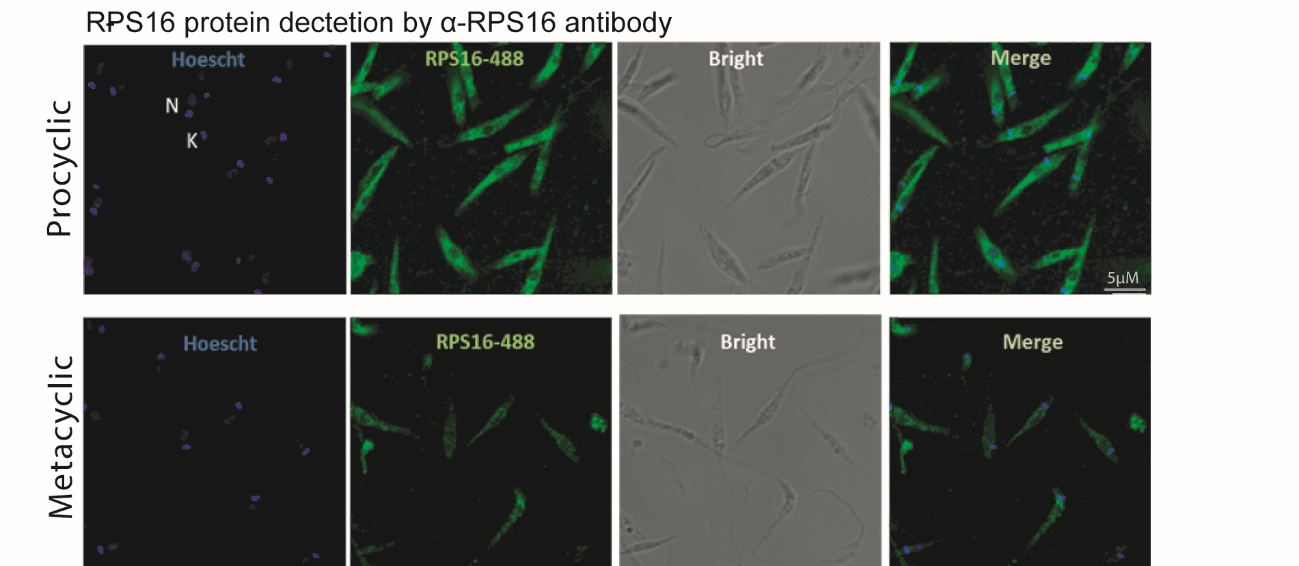

Supplement: S2 Fig — (DOCX) [file pone.0292152.s002.docx]
