# Supplementary material for: Leishmania Ribosomal Protein (RP) paralogous genes compensate each other’s expression maintaining protein native levels
Source: PLoS One. 2024 May 16;19(5):e0292152. doi: 10.1371/journal.pone.0292152 (PMC11098316; doi:10.1371/journal.pone.0292152)
Supplement: S3 Fig — (DOCX) [file pone.0292152.s003.docx]

**S3Fig.** **Confirmation of individual knockouts by conventional PCR for each of the duplicated genes**. All tagged and knockout cell lines were generated using CRISPR/Cas9 genomic editing system, by which the 3xmyc tag sequence was added always at 5’ end of each transcript. (A) Lmj.F.26.0880 transcript was tagged (tag) in the presence (+) and when Lmj.F.26.0890 was knocked out (-). (B) Lmj.F.26.0890 transcript tagged before and after Lmj.F.26.0880 knockout. (C) Lmj.F.15.0200 transcript tagged before and after LmJ.F.34.0860 knockout and (D) the opposite: LmJ.F.34.0860 tagged before and after Lmj.F.15.0200 knockout. General abbreviations: BLAST: Blasticidin-S deaminase resistance gene; 3xmyc*:* 3 times myc epitopes; *PURO:* puromycin resistance gene; Parental cell line (wt); C-: negative control of PCR (reaction with no DNA); M: DNA molecular weight. Arrows represent the primers position used for amplification by PCR. (D) Strategy used for double knockout of RPS16 duplicated genes and the respective sizes for parental and donor DNA sequences. (E) Confirmation of knockout using confirming the presence of the donor insert. However, the presence of (F) Lmj.F.26.0880 and (G) Lmj.F.26.0890 remain genes confirmed the unsuccessful for knockout both genes out.

**
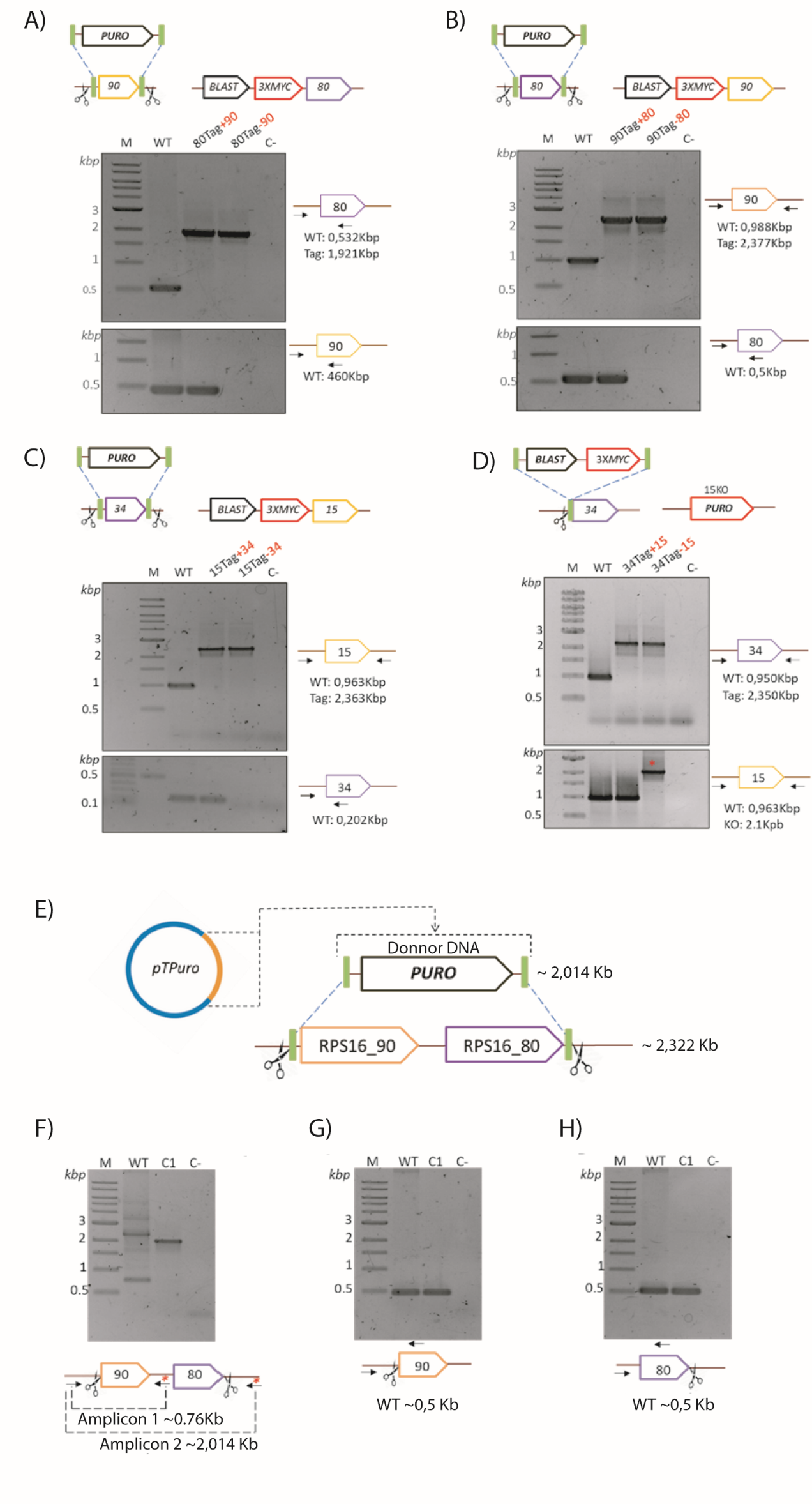
**
