# Supplementary material for: Leishmania Ribosomal Protein (RP) paralogous genes compensate each other’s expression maintaining protein native levels
Source: PLoS One. 2024 May 16;19(5):e0292152. doi: 10.1371/journal.pone.0292152 (PMC11098316; doi:10.1371/journal.pone.0292152)
Supplement: S4 Fig — (DOCX) [file pone.0292152.s004.docx]

**S4Fig.** **Motifs found using the 3’ UTR sequences for all RP transcripts**. Sequences were based on the TriTryp data base, being used for pulldown assay and applied to Multiple Em for Motif Elucidation (MEME - [https://meme-suite.org/meme/tools/meme](about:blank)): (A) three motifs were found present in the 3’UTRs of all four RP genes with similar distribution along the sequences. (B) Motifs exclusively present in the 3’UTR of the high expressed transcripts (RPL13a_15 and RPS16_80) and (C) the respective shared binding proteins identified by *in vitro* pulldown assays. (D) Motifs exclusively present in the 3’UTR of the less expressed transcripts (RPL13a_34 and RPS16_90) for (E) which only two proteins were identified as binding exclusively to these sequences *in vitro*.


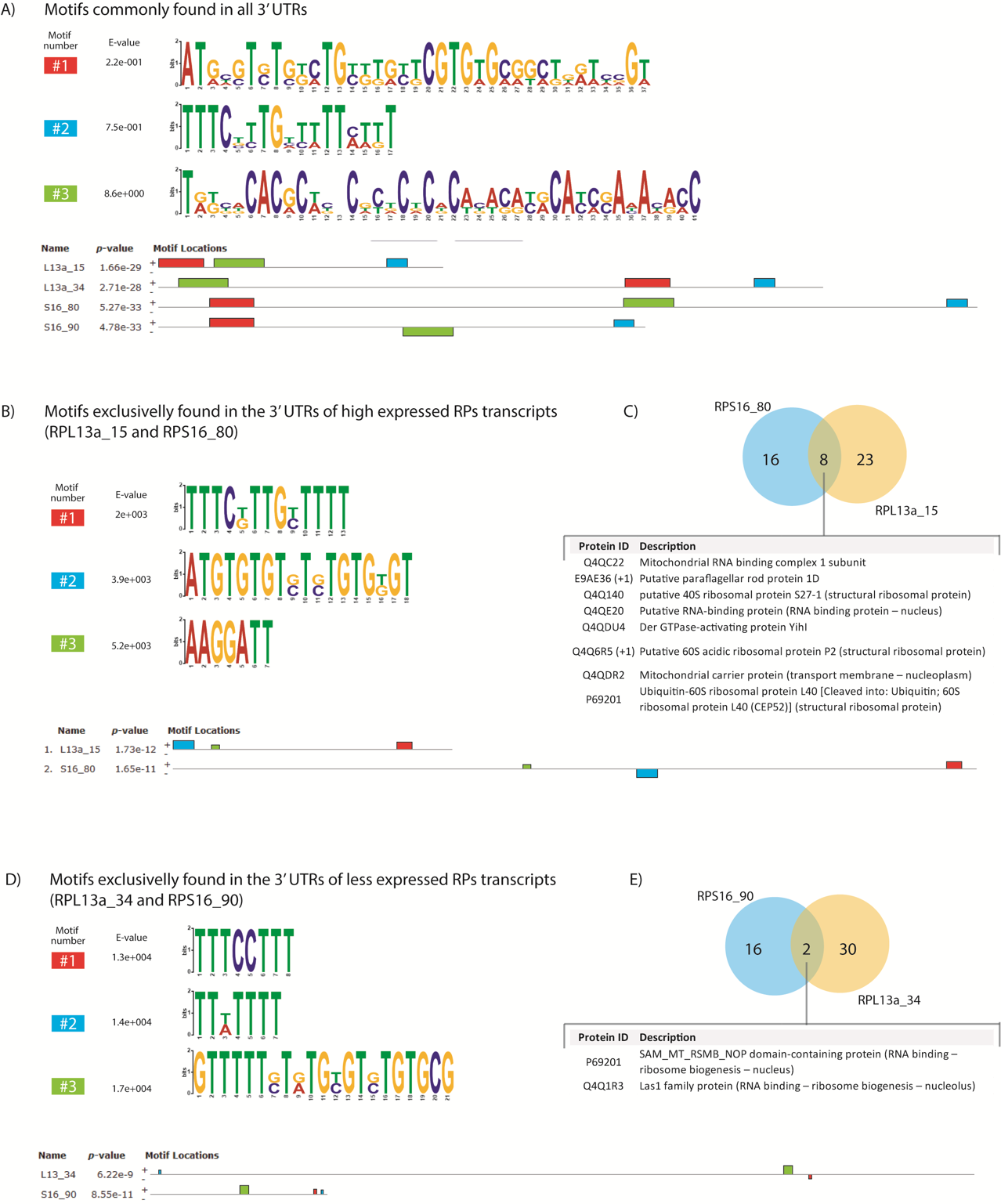
.
