# Supplementary figures and images for: Leishmania Ribosomal Protein (RP) paralogous genes compensate each other’s expression maintaining protein native levels
Source: PLoS One. 2024 May 16;19(5):e0292152. doi: 10.1371/journal.pone.0292152 (PMC11098316; doi:10.1371/journal.pone.0292152)

S5Fig. Full western blotting membranes for all blottings presented in the article.


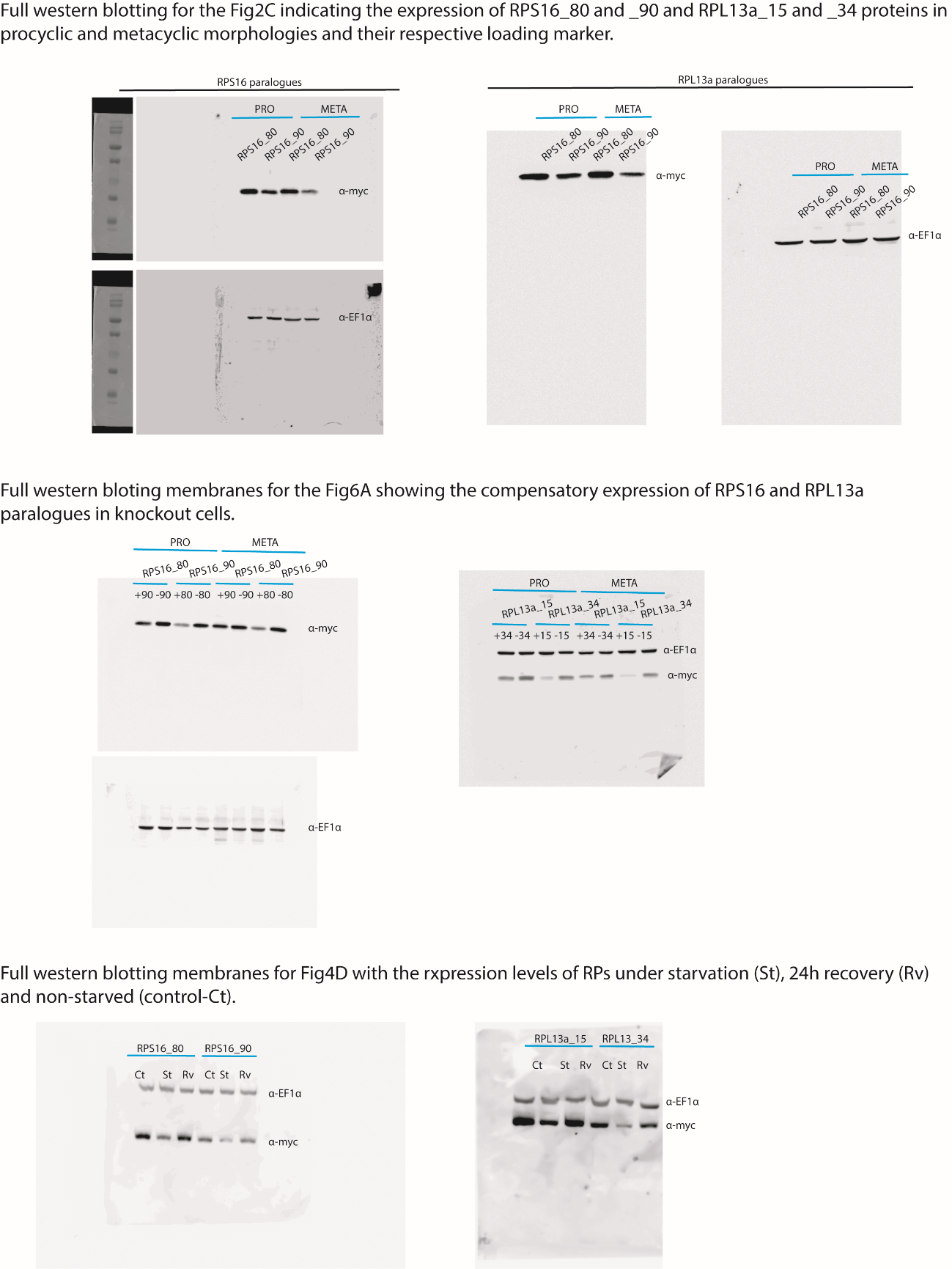


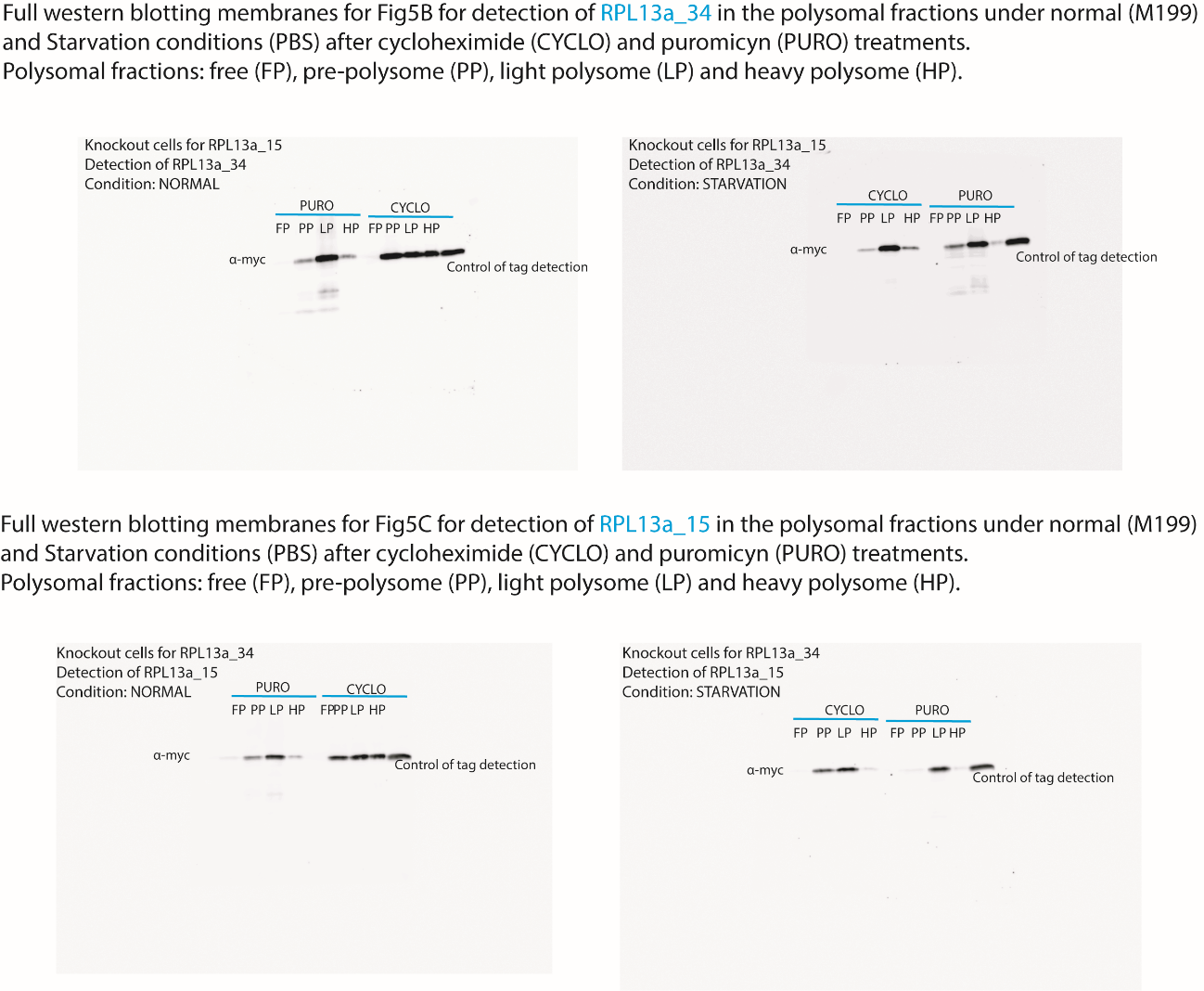

Supplement: S5 Fig — (DOCX) [file pone.0292152.s005.docx]
