# Supplementary material for: Leishmania Ribosomal Protein (RP) paralogous genes compensate each other’s expression maintaining protein native levels
Source: PLoS One. 2024 May 16;19(5):e0292152. doi: 10.1371/journal.pone.0292152 (PMC11098316; doi:10.1371/journal.pone.0292152)

**S6Fig. Sequencing results for all tag insertions.** All RP genes studied here was tagged at 5’-end by CRISPR/Cas9 system with m*yc* sequence (3xMyc) added to each gene. Specific primers were used to amplify the tagged region, from the *myc* sequence to the CDS, by conventional PCR. These amplicons were cloned into a pCR4-TOPO plasmid and sequenced by the Sanger method using M13 primers. Results are shown and confirm the 3x*myc* tag insertion. Primer sequences are shown in the supplementary table 3 (ST3). PGKB5’: 5’-end of the *myc* tag. GS linker: sequence between the tag and tagged gene. The beginning of all coding sequences (CDS) is shown in yellow, and the identity of each gene is indicated.


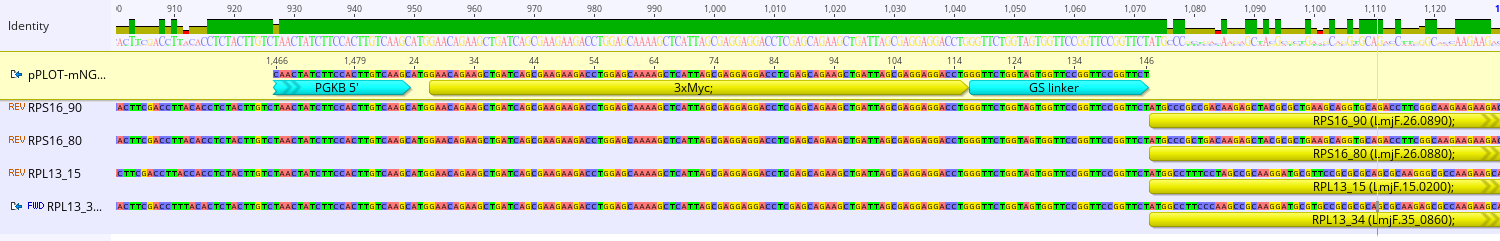

Supplement: S6 Fig — (DOCX) [file pone.0292152.s006.docx]
