# Supplementary material for: Leishmania Ribosomal Protein (RP) paralogous genes compensate each other’s expression maintaining protein native levels
Source: PLoS One. 2024 May 16;19(5):e0292152. doi: 10.1371/journal.pone.0292152 (PMC11098316; doi:10.1371/journal.pone.0292152)
Supplement: S1 Table — (DOCX) [file pone.0292152.s007.docx]

**ST1.** **Proteins binding to the 3’UTRs of RPL13a duplicated genes**. Pulldown assays were performed *in vitro* as described in the methods section, using the sequence for 3’UTR of each RPL13a transcript as RNA template. Proteins specifically binding to these sequences were identified by mass spectrometry.

| **RPL13a_15 – Exclusive Proteins** | | |
| --- | --- | --- |
| LmjF.30.1450 | Q4Q7F9 | Putative kinesin (EC 3.6.4.4) |
| LmjF.25.0430 | Q4QA52 | Guanine nucleotide-binding protein subunit beta-like protein |
| LmjF.03.0230 | E9ACH0 | Putative long chain fatty Acyl CoA synthetase (EC 6.2.1.3) |
| LmjF.21.1620 | Q4QC22 | Mitochondrial RNA binding complex 1 subunit |
| LmjF.29.1270 | E9ADY5 | Putative serine peptidase |
| LmjF.25.1780 | Q4Q9R1 | ATP-grasp domain-containing protein |
| LmjF.30.0580 | Q4Q7P8 | AMP-binding domain-containing protein |
| LmjF.29.1750 | E9AE36 (+1) | Putative paraflagellar rod protein 1D |
| LmjF.29.0030 | E9ADK5 | Ribosomal protein L3-like protein (structural ribosomal protein) |
| LmjF.36.3750 | Q4Q140 | Putative 40S ribosomal protein S27-1 (structural ribosomal protein) |
| LmjF.04.0860 | Q9U0Z7 | Ribosomal_L30 domain-containing protein (mitochondrial large ribosomal subunit) |
| LmjF.13.1520 | Q4QG02 | AAA domain-containing protein |
| LmjF.19.1230 | Q4QD92 | Uncharacterized protein |
| LmjF.21.0530 | Q4QCG2 | TPR_REGION domain-containing protein |
| LmjF.21.0750 | Q4QCD8 | Uncharacterized protein |
| LmjF.18.0220 | Q4QE20 | Putative RNA-binding protein (RNA binding protein – nucleus) |
| LmjF.28.0930 | Q4Q8H4 | Archaic_translocase_outer_mitochondrial_membrane_ 40_-_putative |
| LmjF.18.0980 | Q4QDU4 | Der GTPase-activating protein YihI |
| LmjF.30.3730 | Q4Q6R5 (+1) | Putative 60S acidic ribosomal protein P2 (structural ribosomal protein) |
| LmjF.18.1280 | Q4QDR2 | Mitochondrial carrier protein (transport membrane – nucleoplasm) |
| LmjF.24.0990 | Q4QAN4 | Putative replication factor C, subunit 1 (DNA replication factor) |
| LmjF.34.2610 | Q4Q2T4 | RuvB-like helicase (EC 3.6.4.12) |
| LmjF.03.0190 | E9ACG6 | Putative U2 splicing auxiliary factor (RNA binding protein – nucleus) |
| LmjF.07.0990 | Q4QII8 | Putative nucleolar RNA-binding protein (RNA binding protein – nucleus) |
| LmjF31.1900; LmjF31.2030 | P69201 | Ubiquitin-60S ribosomal protein L40 [Cleaved into: Ubiquitin; 60S ribosomal protein L40 (CEP52)] (structural ribosomal protein) |
| LmjF.08.0320 | Q4QID7 | Putative mitochondrial associated ribonuclease |
| LmjF.32.1070 | Q4Q5H5 | Putative small nuclear ribonucleoprotein (mRNA metabolic process – nucleus) |
| LmjF.18.0830 | Q4QDV9 | Guanine nucleotide-binding protein subunit beta-like protein (ribosome biogenesis – nucleus and ribosome) |
| LmjF.34.0740 | Q4Q3D2 | CBF domain-containing protein (ribosome biogenesis – nucleus and ribosome) |
| LmjF.13.1000 | Q4QG54 | Uncharacterized protein |
| **RPL13a_34 – Exclusive Proteins** | | |
| LmjF.15.1010 | Q4QF83 | NAD-specific glutamate dehydrogenase (EC 1.4.1.2) |
| LmjF.26.0010 | Q4Q9J0 | Ribosome production factor 2 homolog (Ribosome biogenesis protein RPF2 homolog) (ribosome biogenesis – nucleus) |
| LmjF.05.0080 | Q4QJJ5 | Rrp15p-domain-containing protein (ribosome biogenesis) |
| LmjF.34.1000 | Q4Q3A5 | Putative myosin IB heavy chain |
| LmjF.02.0090 | E9AC84 | Uncharacterized protein |
| LmjF.02.0670 | E9ACE1 | Putative mitochondrial carrier protein (mitochondrial transporter) |
| LmjF.28.1440 | Q4Q8C2 | Uncharacterized protein |
| LmjF.21.0490 | Q4QCG6 | Putative DnaJ protein (protein folding process – nucleoplasm and mitochondria) |
| LmjF.10.1160 | Q4QH98 | Putative rab1 small GTP-binding protein |
| LmjF.27.0450 | E9AD22 | Ribosome biogenesis protein (RNA binding protein) |
| LmjF.26.2020 | Q4Q8Z0 | 40S ribosomal protein S30 (ribosome) |
| LmjF.28.1740 | Q4Q892 | SAM_MT_RSMB_NOP domain-containing protein (ribosome biogenesis) |
| LmjF.24.0770 | Q4QAQ6 | Putative malic enzyme (EC 1.1.1.38) |
| LmjF.35.5260 | E9AFZ9 | c-Myc-binding family protein (transcription regulator activity – nucleus) |
| LmjF.32.1802 | Q4Q599 | Uncharacterized protein |
| LmjF.28.2570 | Q4Q804 | Putative splicing factor 3B subunit 1 (mRNA metabolic process – nucleus) |
| LmjF.35.2210; LmjF.35.2220 | E9AF45 | Kinetoplast membrane protein 11 (Kinetoplastid membrane protein-11) |
| LmjF.27.1730 | E9ADF0 | BAR domain-containing protein |
| LmjF.34.0010 | Q4Q3K8 | Short-chain dehydrogenase |
| LmjF.28.0600 | Q4Q8L0 | H15 domain-containing protein (hypothetical protein) |
| LmjF.16.0190 | Q4QF01 | Las1 family protein (ribosome biogenesis – nucleus) |
| LmjF.22.0500 | Q4QBU6 | TFIIS N-terminal domain-containing protein (poly-A mRNA transporter from nucleus) |
| LmjF.36.3950 | Q4Q120 | Coiled-coil domain-containing protein 22 homolog |
| LmjF.33.0970 | Q4Q494 | Uncharacterized protein |
| LmjF.36.6050 | Q4Q0F5 | Uncharacterized protein |
| LmjF23.0270; LmjF.23.0270 | Q01782 | Pteridine reductase 1 (EC 1.5.1.33) (H region methotrexate resistance protein) |
| LmjF.23.1550 | Q4QAY3 | Pre-mRNA-splicing factor SYF1 (mRNA metabolic process – nucleus) |
| LmjF.21.1500 | Q4QC45 | RNA-directed RNA polymerase (hypothetical protein) |
| LmjF.28.2110 | Q4Q854 | CULLIN_2 domain-containing protein |
| LmjF.15.0810 | Q4QFA4 | Peroxisomal membrane protein PEX16 |
| LmjF.29.0140 | E9ADL6 | Plastid-encoded RNA polymerase subunit alpha (transcription) |
| **RPL13a_15 and 34 – Shared Proteins** | | |
| LmjF.18.1610 | Q4QDM8 | Noc2-domain-containing protein (ribosome biogenesis – nucleus) |
| LmjF.18.0740 | Q4QDW8 | Putative elongation factor Tu (translational regulatory activity) |
| LmjF.04.1160 | O97193 | fructose-bisphosphatase (EC 3.1.3.11) |
| LmjF.14.1100 | Q4QFM4 | Putative kinesin K39 |
| LmjF.05.0460 | Q4QJF6 | Nucleolar GTP-binding protein 2 (mitochondria) |
| LmjF.19.0240 | Q4QDJ7 | GN3L_Grn1 domain-containing protein |
| LmjF.27.1380; LmjF.27.1390 | E9ADB9 | 60S acidic ribosomal protein P0 (Ribosomal RNA binding protein) |
| LmjF.09.0940 | Q4QHT0 | NFACT-R_1 domain-containing protein (translational associated element) |
| LmjF.03.0900 | E9ACN5 | Putative peter pan protein (Ribosomal RNA binding protein) |
| LmjF.31.2250 | Q4Q636 | Putative 3,2-trans-enoyl-CoA isomerase,mitochondrial (EC 5.3.3.8) (lipid metabolic process – mitochondria) |
| LmjF.33.0318; LmjF.33.0330 | Q4Q4I0 (+1) | Heat shock protein 83-1 |
| LmjF.28.2260 | Q4Q838 | Putative glycosomal membrane protein |
| LmjF.32.1000 | Q4Q5I2 | T-complex protein 1 subunit epsilon (CCT-epsilon) (protein folding) |
| LmjF.21.1820; LmjF.36.1925 | Q4QC01 | 60S ribosomal protein L37a (Putative 60S ribosomal protein L37a) (Structural ribosomal protein) |
| LmjF.05.0150 | Q4QJI8 | Leucine-rich repeat protein |
| LmjF.15.0330 | Q4QFE7 | Abhydrolase_3 domain-containing protein |
