# Supplementary material for: Leishmania Ribosomal Protein (RP) paralogous genes compensate each other’s expression maintaining protein native levels
Source: PLoS One. 2024 May 16;19(5):e0292152. doi: 10.1371/journal.pone.0292152 (PMC11098316; doi:10.1371/journal.pone.0292152)
Supplement: S2 Table — (DOCX) [file pone.0292152.s008.docx]

**ST2.** **Proteins binding to the 3’UTRs of RPS16 duplicated genes**. Pulldown assays were performed *in vitro* as described in the methods section, using the sequence for 3’UTR of each RPS16 transcript as RNA template. Proteins specifically binding to these sequences were identified by mass spectrometry.

| **RPS16_90 – Exclusive Proteins** | | |
| --- | --- | --- |
| LmjF.22.0210 | Q4QBX5 | CBF domain-containing protein (nuclear protein 168) |
| LmjF.14.1100 | Q4QFM4 | Putative kinesin K39 |
| LmjF.25.0430 | Q4QA52 | Guanine nucleotide-binding protein subunit beta-like protein (RNA binding – ribosome biogenesis – ribosome) |
| LmjF.28.1080 | Q4Q8F9 | HMG box domain-containing protein (mitochondrial) |
| LmjF.11.1000 | Q4QGX9 | Pyruvate, phosphate dikinase (EC 2.7.9.1) |
| LmjF.35.3860 | E9AFL0 | CCT-eta (protein folding) |
| LmjF.18.1370 | Q4QDQ2 | Putative heat shock protein |
| LmjF.11.0410 | Q4QH39 | Ribosome biogenesis protein BOP1 homolog (RNA binding – ribosome biogenesis – ribosome) |
| LmjF.16.1425; LmjF.16.1427; LmjF.16.1430 | Q4QEM2 | Paraflagellar rod protein 2C |
| LmjF.24.1640 | Q4QAG7 | Fatty acyl-CoA reductase (EC 1.2.1.84) |
| LmjF.28.1740 | Q4Q892 | SAM_MT_RSMB_NOP domain-containing protein (RNA binding – ribosome biogenesis – nucleus) |
| LmjF.31.0190 | Q4Q6P5 | Putative nucleolar protein (RNA binding – ribosome biogenesis – nucleolus) |
| LmjF.16.0190 | Q4QF01 | Las1 family protein (RNA binding – ribosome biogenesis – nucleolus) |
| LmjF.30.0120 | Q7YWB6 | Alkyl dihydroxyacetonephosphate synthase (Alkyldihydroxyacetonephosphate synthase (EC 2.5.1.26)) |
| LmjF.32.1070 | Q4Q5H5 | Putative small nuclear ribonucleoprotein (mRNA metabolic – nucleus) |
| LmjF.17.0760 | Q4QEC8 | AB hydrolase-1 domain-containing protein |
| LmjF.10.0910 | Q4QHC3 | Putative small GTP-binding protein Rab11 |
| LmjF.31.1220 | Q4Q6E1 | H(+)-exporting diphosphatase (EC 7.1.3.1) |
| **RPS16_80 – Exclusive Proteins** | | |
| LmjF.18.0620; LmjF.36.3760 | Q4QDX9 | Putative 60S ribosomal protein L10a (ribosome constituint) |
| LmjF.14.0150 | Q4QFX0 | Putative 60S ribosomal protein(ribosome constituint) |
| LmjF.02.0090 | E9AC84 | Uncharacterized protein |
| LmjF.09.0940 | Q4QHT0 | NFACT-R_1 domain-containing protein (translational associated element) |
| LmjF.02.0670 | E9ACE1 | Putative mitochondrial carrier protein |
| LmjF.25.1540 | Q4Q9T7 | Mitochondrial RNA binding complex 1 subunit |
| LmjF.21.1620 | Q4QC22 | Mitochondrial RNA binding complex 1 subunit |
| LmjF.21.0490 | Q4QCG6 | Putative DnaJ protein (protein folding process – nucleoplasm and mitochondria) |
| LmjF.10.1160 | Q4QH98 | Putative rab1 small GTP-binding protein |
| LmjF.29.1750 | E9AE36 (+1) | Putative paraflagellar rod protein 1D |
| LmjF.25.2130; LmjF.25.2140 | Q4Q9M4 | Succinate--CoA ligase [ADP-forming] subunit alpha, mitochondrial (EC 6.2.1.5) (Succinyl-CoA synthetase subunit alpha) (SCS-alpha) |
| LmjF.36.3750 | Q4Q140 | Putative 40S ribosomal protein S27-1 (ribosome constituint) |
| LmjF.35.5260 | E9AFZ9 | c-Myc-binding family protein (transcription regulator activity – nucleus) |
| LmjF.11.1360 | Q4QGU6 | Ribosomal protein L4 (ribosome constituint) |
| LmjF.18.0220 | Q4QE20 | Putative RNA-binding protein 1 (mRNA metabolic process – nucleus) |
| LmjF.18.0980 | Q4QDU4 | Der GTPase-activating protein YihI |
| LmjF.30.3730 | Q4Q6R5 (+1) | Putative 60S acidic ribosomal protein P2 (structural ribosomal protein) |
| LmjF.18.1280 | Q4QDR2 | Mitochondrial carrier protein |
| LmjF.35.2210; LmjF.35.2220 | E9AF45 | Kinetoplast membrane protein 11 (Kinetoplastid membrane protein-11) |
| LmjF.21.1820; LmjF.36.1925 | Q4QC01 | 60S ribosomal protein L37a (Putative 60S ribosomal protein L37a) (Structural ribosomal protein) |
| LmjF.27.1730 | E9ADF0 | BAR domain-containing protein |
| LmjF.31.1900; LmjF.31.2030 | P69201 | Ubiquitin-60S ribosomal protein L40 [Cleaved into: Ubiquitin; 60S ribosomal protein L40 (CEP52)] (structural ribosomal protein) |
| LmjF.23.0270 | Q01782 | Pteridine reductase 1 (EC 1.5.1.33) (H region methotrexate resistance protein) |
| LmjF.36.1620 | Q4Q1R3 | Universal minicircle sequence binding protein (translational regulatory activity) |
| **RPS16_90 and 80 – Shared Proteins** | | |
| LmjF.18.1610 | Q4QDM8 | Noc2-domain-containing protein (ribosome biogenesis – nucleus) |
| LmjF.30.1450 | Q4Q7F9 | Putative kinesin (EC 3.6.4.4) |
| LmjF.19.1560 | Q4QD53 | Inosine-5'-monophosphate dehydrogenase (IMP dehydrogenase) (IMPD) (IMPDH) (EC 1.1.1.205) |
| LmjF.05.0080 | Q4QJJ5 | Rrp15p-domain-containing protein (ribosome biogenesis) |
| LmjF.27.1380; LmjF.27.1390 | E9ADB9 | 60S acidic ribosomal protein P0 (Ribosomal RNA binding protein) |
| LmjF.03.0900 | E9ACN5 | Putative peter pan protein (Ribosomal RNA binding protein) |
| LmjF.31.2250 | Q4Q636 | Putative 3,2-trans-enoyl-CoA isomerase,mitochondrial (EC 5.3.3.8) |
| LmjF.33.0318; LmjF.33.0330 | Q4Q4I0 (+1) | Heat shock protein 83-1 |
| LmjF.28.2260 | Q4Q838 | Putative glycosomal membrane protein (peroxisome) |
| LmjF.21.0100 | Q4QCK6 | Uncharacterized protein |
| LmjF.28.1010; LmjF.28.1030 | Q4Q8G4 | Putative ribosomal protein S20 (structural ribosomal protein) |
