# Supplementary material for: Leishmania Ribosomal Protein (RP) paralogous genes compensate each other’s expression maintaining protein native levels
Source: PLoS One. 2024 May 16;19(5):e0292152. doi: 10.1371/journal.pone.0292152 (PMC11098316; doi:10.1371/journal.pone.0292152)
Supplement: S3 Table — (DOCX) [file pone.0292152.s009.docx]

**ST3.** **Primers used for all transfections**. 5’🡪3’ sequences for the primers used in this work for knockout and tagging of the RP genes.

| **Primer ID** | **Sequence 5’🡪3’** | **Application** |
| --- | --- | --- |
| G00_SG_Scaffold | AAAAGCACCGACTCGGTGCCACTTTTTCAAGTTGATAACGGACTAGCCTTATTTTAACTTGCTATTTCTAGCTCTAAAAC | Single guide (sg) DNA generation by PCR for transfection and tagging of RP genes |
| LmjF26.0880_sg1 | gaaattaatacgactcactataggCTGAGTTGGAGATGCAAGAAgttttagagctagaaatagc |  |
| LmjF26.0890_sg1 | gaaattaatacgactcactataggTGATGCTTTCACTCTAACCGgttttagagctagaaatagc |  |
| LmjF.15.0200_SG_5' | GAAATTAATACGACTCACTATAGGCTAGGAAAGGCCATTTTTCAGTTTTAGAGCTAGAAATAGC |  |
| LmjF.34.0860_SG_5' | GAAATTAATACGACTCACTATAGGCAATTGAAGCATGCAAAGGCGTTTTAGAGCTAGAAATAGC |  |
| LmjF26.0880_tagF | CTAACGCGCCAATATGCCCATGTATATCCCgtataatgcagacctgctgc | Donor DNA generation by PCR used as selection markers for tagging of the RP genes |
| LmjF26.0880_90_tagR_3myc_only | CTTCAGCGCGTAGCTCTTGTCAGCGGGCATAGAACCGGAACCGGAAC |  |
| LmjF26.0890_tagF | CGCGTGTGCGCATCCATCGTGCAGTCACCCgtataatgcagacctgctgc |  |
| LmjF26.0880_90_tagR_3myc_only | CTTCAGCGCGTAGCTCTTGTCAGCGGGCATAGAACCGGAACCGGAAC |  |
| LmjF.15.0200_myctag_F | CTGTTTTACGACTCCCCTCCCACACCGTGAGTATAATGCAGACCTGCTGC |  |
| LmjF.15.0200_myctag_R | GAACGCATCCTTGCGGCTAGGAAAGGCCATAGAACCGGAACCGGAAC |  |
| LmjF.34.0860_myctag_F | TCCCTTTCACCTCATCGCACGCACGCTCCTGTATAATGCAGACCTGCTGC |  |
| LmjF.34.0860_myctag_R | GCACGCATCCTTGCGGCTTGGGAAGGCCATAGAACCGGAACCGGAAC |  |
| G00_SG_Scaffold | AAAAGCACCGACTCGGTGCCACTTTTTCAAGTTGATAACGGACTAGCCTTATTTTAACTTGCTATTTCTAGCTCTAAAAC | Single guide (sg) DNA generation by PCR for transfection and knockout of the RP genes |
| SG_5'_tagMYC* | gaaattaatacgactcactataggCTGTGCCCCTAGCTTGCAGCgttttagagctagaaatagc |  |
| SG_3'_S16_80_R | gaaattaatacgactcactataggTACGATTGCGCGAGTGACTGgttttagagctagaaatagc |  |
| SG_3'_S16_90_R | gaaattaatacgactcactataggATGCACCTCGCACGCTGCTTgttttagagctagaaatagc |  |
| SG_3'_L13_15_R | gaaattaatacgactcactataggTGGGTTCGTGAGAAGCGAGAgttttagagctagaaatagc |  |
| SG_3'_L13_34_R | gaaattaatacgactcactataggATCGACACGACCACCCTGCAgttttagagctagaaatagc |  |
| LmjF26.0880_tagF | CTAACGCGCCAATATGCCCATGTATATCCCgtataatgcagacctgctgc | Donor DNA generation by PCR used as selection markers for transfection and knockout of the RP genes |
| S16_80_KO_R | CGCGTGTGAGGGTTACGATTGCGCGAGTGAccaatttgagagacctgtgc |  |
| LmjF26.0890_tagF | CGCGTGTGCGCATCCATCGTGCAGTCACCCgtataatgcagacctgctgc |  |
| S16_90_KO_R | CTGCCATGCACGATCTCTCCACATCCTAAGccaatttgagagacctgtgc |  |
| LmjF.15.0200_myctag_F | CTGTTTTACGACTCCCCTCCCACACCGTGAGTATAATGCAGACCTGCTGC |  |
| L13_15_KO_R | GAGAGACAGCGTGAAACAGTAGACAATCCTccaatttgagagacctgtgc |  |
| LmjF.34.0860_myctag_F | TCCCTTTCACCTCATCGCACGCACGCTCCTGTATAATGCAGACCTGCTGC |  |
| L13_34_KO_R | TTCCAACAACGTCATGACGGCTAAGAACCGccaatttgagagacctgtgc |  |
| S16_80_KO/tag_confirm_F | ATCCTTTCGTTTTAGGCCCATC | Confirmation of tagging and KO |
| S16_80_KO/tag_confirm_R | CACCGTTCACCTTGATGTTGC |  |
| S16_90_KO/tag_confirm_F | GTTAGAGTGAAAGCATCAGCCC |  |
| S16_90_KO/tag_confirm_R | GTGCGGAACAAAATAATGTCAG |  |
| confirm_KO_por_ausência_S16_80_90_R | CACCGTTCACCTTGATGTTGC |  |
| L13_15_confirm_tag_KO_F | AACCTCTCCAAACACACGCA |  |
| L13_15_confirm_Tag_KO_R | ACCATCACCGCCGAAGAGAA |  |
| L13_34_confirm_Tag/KO_F | TCCCCTTTCCACCTCCATCT |  |
| L13_34_confirm_Tag/KO_R | ACTCTCAATTCTCGTTCGCC |  |
| confirm_KO_por_ausência_15_34_R | CACGTGATCCTTCAGGTCGA |  |

| RPS16_Rv | CACCGTTCACCTTGATGTTGC | Align inside the CDS of both S16 genes for 5’UTR amplification |
| --- | --- | --- |
| RPS16_80_Fw | CATGCGCCATCCTTGTCATG | Specific alignment to the 5’UTR |
| RPS16_90_Fw | TTGCGTATCCATTACCCGTG |  |
| RPL13_Rv | CAATGTTGAGCTGCTCGCAG | Align inside the CDS of both S16 genes for 5’UTR amplification |
| RPL13_15_Fw | AACCTCTCCAAACACACGCA | Specific alignment to the 5’UTR |
| RPL13_34_Fw | TTCCAACAACGTCATGACGGCTAAGAACCG |  |
