# Supplementary material for: Leishmania Ribosomal Protein (RP) paralogous genes compensate each other’s expression maintaining protein native levels
Source: PLoS One. 2024 May 16;19(5):e0292152. doi: 10.1371/journal.pone.0292152 (PMC11098316; doi:10.1371/journal.pone.0292152)
Supplement: Attachment — Submitted filename: Response to Reviewers.docx [file pone.0292152.s011.docx]

We thank the reviewers for the constructive criticism and comments that improved the manuscript and its contribution.

**Reviewer #1:**

The manuscript entitled "Leishmania Ribosomal Protein (RP) paralogous genes compensate each other’s expression maintaining protein native levels", is a very compact and elegant work that sheds light on the mechanisms of gene expression in trypanomatids, particularly in Leishmania. However, the manuscript can be improved in its presentation.

Some suggestions are:

1. Although the authors state that the study focuses on the role of different UTRs in regulation (lines 255-259), rather than function, since approximations are made to the possible non-canonical functions of the ribosomal genes under study (nutritional stress assays), it is suggested:

- Point out the a.a. that change between copies of RPL13a in the Introduction.

This information was added to the Introduction (lines 93-96) – “RPS16 proteins are identical while for L13a isoforms, a Phenylalanine (Phe10) and a Glycine (Gly16) present in L13a_15 are respectively replaced in the L13a_34 by Cysteine (Cys) and Serine (Ser), both subject to post-translational modifications (PTMs).”

- To perform *in silico* models of the three-dimensional structure of RPL13a in order to discard possible structural changes affecting the protein function.

As per reviewer #1 suggestion we performed in silico models using Alphafold and isoforms of RPL13a three dimensional structures suggest that protein structures are identical (SFig1D).

2. Avoid repeating the information provided to the reader in the Introduction in the Discussion.

Where in the text we detected repeated information, we deleted or modified the text accordingly. These modifications are indicated in the version with track changes.

3. The results concerning the Cis elements found in the UTRs are very important and, in fact, are included in the abstract. therefore, I suggest including them in Materials and methods and results and including the figure in the body of the manuscript.

We present these results as supplementary material because despite its putative relevance, we did not find statistically significant E-values (< 0.05), as indicated in the supplementary figure. In the revised manuscript we included the information in both methods and results sections. Nevertheless, given the significance of the result we maintained its representation in the supplementary material.

4. Lines 328 to 331 correspond more to the discussion than to the results.

The sentence below was removed from the results section. We did not transfer the paragraph to the discussion to avoid redundancy, since similar content was already included in the discussion.

5. Clarify the stage of the parasites in Figure 3C.

Parasite morphology information was added in the Figure subtitle.

6. RT-qPCR results of expression of RPS16 80 and 90 paralogs in mutant vs. wild-type parasites are not shown.

These assays were not performed since the CDSs are identical. We tested different combinations of primers but the few ones that could distinguish RPS16 80 from 90 did not amplify within an acceptable range.

7. As for the discussion, I suggest changing the order. I mean, after presenting the two possible reasons for the parasite to have the same gene with two equal UTRs (lines 465 to 468), continue with the regulation issues, then the functionality, to finish with the compensatory effect.

We accepted the reviewer’s suggestion, and the order was modified in the results and discussion. Thus, the items in the results were switched as blocks. For the sake of clarity, we did not highlight the block switches, but only the altered texts within these blocks.

Original order:

1. RPs from paralog genes have different expression levels and undistinguishable subcellular distribution
2. Duplicated RPs present a paralog compensation that maintains protein levels
3. Profile of proteins interacting with the UTRs of the studied RP transcripts
4. Increased response to starvation and RP levels of mutant parasites
5. RPL13a accumulates in non-polysomic fractions under starvation

New order:

1. RPs from paralog genes have different expression levels and undistinguishable subcellular distribution
2. Profile of proteins interacting with the UTRs of the studied RP transcripts
3. Increased response to starvation and RP levels of mutant parasites
4. RPL13a accumulates in non-polysomic fractions under starvation
5. Duplicated RPs present a paralog compensation that maintains protein levels

8. Regardless of the stage, procyclic or metacyclic promastigotes, there is one copy of both genes that is expressed at the protein level more than the other. Could the length of the corresponding UTRs have something to do with it?

We found no correlation between 3’UTR length and levels of expression of corresponding proteins. In fact, L13a-34 and S16-90 are the ones presenting lower levels of expression compared to their paralogous copies, but S16-90 presents the shorter 3’UTR and L13a-34, the longest, as compared to the paralogous copy.

9.I wonder what the authors think about what would happen in the amastigote stage in relation to their research questions. I suggest including in the discussion.

Amastigotes, as procyclics, are host-specific replicative forms. Therefore, it is expected that their translational machineries are very active. Thus, we believe that the levels and the compensatory mechanism for these isoforms in amastigote will resemble those observed in procyclics, but at this point this is hypothetical. Notwithstanding relevant, we have experimental limitations for cultivation of *L. major* axenic amastigotes *in vitro,* being this the reason to restrict the study to the promastigote stages.

10. It would be very interesting for the authors to discuss the possible effect of mutations at the level of promastigote replication, metacyclogenesis, infective capacity and amastigote replication.

Apart from the nutritional stress, for its potential correlation with ribosome and translation activity, we evaluated the polysome profile differences. We extracted metacyclics but did not conduct an analysis of metacyclogenesis neither infectivity or amastigote replication.

11. Revise figures and supplementary material carefully as there are changes in gene or protein names, double legends, etc.

We noticed that the description of figure 3A might not be clear to the reader, so we modified the description of the cartoon (lines 353-358).

Another aspect that may have caused confusion is the Supplementary figure 5. We included SF5 as supplementary material, because all the membranes presented in the article were strips cropped from the entire membranes in all the figures mentioned in SF5. Thus, the aim of SF5 is to show that there are no hidden signals/bands, no manipulation of results obtained.

12. Include the database to which readers can refer to look up the raw data from the study, especially the pull-down findings.

The file with all pull-down results was added to supplementary material. Raw data is in the process of deposit in the ProteomicXchange repository.

**Reviewer #2:**

Many ribosomal protein (RP) genes are duplicated in Leishmania. Dr Angela Cruz and coworkers have investigated the expression of the paralogous pairs of RP RPS16 and RPL13a during the transition from normal growth to starvation conditions. They report that the deletion of one of the two paralogous genes leads to increased protein expression from the other, though the corresponding change in mRNA levels is not observed. This agrees with the current model that most regulation of gene expression is post-transcriptional in Leishmania. Interestingly, microscopic experiments indicate that cytoplasmic r-proteins are distributed in several foci, which do not change during the transition from active growth to starvation. Finally, they analyze the ribosome organization through polysome gradient experiments. A peak sedimenting a little faster than the 80S is interpreted to represent a non-ribosomal RP-mRNA complex. The problem is interesting, but several aspects of the work need further investigation prior to publication.

Major issues

• Please mention the names of the RPs in the universal nomenclature (<http://dx.doi.org/10.1016/j.sbi.2014.01.002>) in order to facilitate comparison to results with other species.

We would like to justify not using the mentioned universal nomenclature on the original version. On the system proposed by Ban et al (2014), homologous ribosomal proteins are assigned the same name, regardless of species. They organized the nomenclature of ribosomal proteins to make it universal and easily recognizable irrespective of the origin. Nevertheless, Ban’s proposed nomenclature has not been broadly adopted by the community, and this was the reason for not employing it. More importantly, the nomenclature used in *Leishmania* is not the universal one, and we opted to use the nomenclature of the trypanosomatids annotated genomes, otherwise we would only increase entropy and confusion within the *Leishmania* community. Therefore, we added a brief explanation of the nomenclature issue, making the correlation of the *Leishmania* ribosomal protein identification with the corresponding Ban’s universal ones (L13a corresponds to uL13 and S16, to uS9).

In the revised version of the manuscript, this information has been added to Materials and Methods (lines 108-112).

• There are several problems with the polysome experiment (Figure 6)
o Show the OD trace from active growth and starvation conditions on comparative scales to allow a (semi-)quantitative comparison of the peaks.

As suggested by the reviewer, we have modified Figure 6A by inserting the scale with arbitrary values of A254 nm, since the ISCO instrument does not provide absolute OD values. To assign these values, we relied on the centimetre scale of the ISCO paper on which the gradients are plotted. Considering that the gradients were made based on the number of cells (5 x10^8^), with the same sensitivity in the instrument (1.0) and starting the gradients at the baseline of 30 cm on the ISCO paper, we believe that our strategy for defining the scale for a semi-quantitative analysis is consistent with the reviewer's suggestion. In addition, we estimated the absorbance values to define the reduction of the 60S and 80S peaks relative to the profile of the parental strain. All these modifications are described in the Materials and Methods section and in the legend of Figure 6.

o The peak moving slightly faster than the 80S particles runs similarly to the half-mers found in several other species. The authors should do a northern analysis of the fractions, at least in the 80S-disome range. If the authors’ interpretation is correct, no rRNA peak corresponding to the OD trace should be found between the 80S and the disome.

We agree with the reviewer's observation that the peak moving slightly faster than 80S may resemble a halfmer. However, we do not state in the article that it is a disome or a halfmer. In fact, knockout of RPL13a paralogs caused a decrease in the 60S ribosomal subunit and consequently a reduction in the 80S monosome and polysomes, as observed for other ribosomal proteins in some organisms, suggesting a canonical role for RPL13a as a ribosomal protein. Although the polysomal profile of mutants from several different organisms knocked out for ribosomal proteins of the 60S subunit showed halfmers, we did not observe this similarity in the peaks related to polysomes in our gradients. Furthermore, when we looked at the disome peak in the gradient of the parental strain (where both copies of RPL13a are present), we observed the same distribution pattern for both mutants (Δ RPL13a_15 and Δ RPL13a_34). Regarding the definition of the area between 80S and disome, it should be noted that this technique does not have sufficient resolution to separate these fractions. Therefore, it would be difficult to obtain an rRNA-free fraction. In the article by Anish et al. (RNA (2012), 18:1968-1983), it is possible to verify the presence of rRNA in the range between 80S and disome, which supports our justification.

• The analysis of proteins binding to the 3’ORF should be continued with washes of incremental salt concentration to determine the hierarchy of affinities.

We thank the reviewer for raising this point, as it would be a thorough route to monitor the affinity force between proteins and UTRs. Such assay would be a step forward to improve our understanding of the putative interactions, along with RIP using tagged proteins, and other complementary assays for the analysis of RNA-protein interactions; they should improve precision to identify hierarchically protein binding profiles to distinct UTRs, but it is our understanding that these approaches should compose another study and are not on the scope of the present investigation. In the current study, and on the specific matter, our aim was to identify distinct protein profiles bound to the paralogous’ UTRs that could reinforce the hypothesis that these players, RBPs and UTRs, are in the core of the differential expression observed for the paralogous copies of the studied genes. Therefore, the affinity evaluation and complementary approaches as such abovementioned should be the object of a subsequent investigation.

• Line 44: the statement is too general. The are many scries without such gene duplication, e.g., K. lactis and C albicans.

The paragraph has been modified to add precision (lines 43 to 50), specifically mentioning *S. cerevisiae*. In addition, a text on *S. cerevisiae* duplicated RP genes has been inserted in the first paragraph of Discussion.

• The writing style should be tightened. The current version is quite conversational, at places it goes on extensive excursions for which there is little rigorous evidence (e.g., lines 598-603).

We worked on the writing style aiming to improve precision and objectivity. The mentioned text at lines 598-603 have modified excluding fragments too speculative or misleading.

• Lines 197-198: samples did not enter the separation gel? How were the proteins then fractionated?

For the mass spectrometry identification, the proteins were not fractionated in the resolving layer of the polyacrylamide gel, the electrophoresis is run to allow proteins to cross the stacking layer and line up only entering the resolving layer, to be collected as a unique band with all proteins. The in-gel proteins were sent to analysis in the Proteomics Platform of Laval University, Quebec, Canada, responsible for the sample preparation and peptide identification, as described. As previously mentioned, RAW Data is in the process of deposit in the ProteomeXchange repository.

Minor issues

• The English language is generally good, but a review of the use of articles is suggested.

The manuscript was sent to an expert for improving English language as suggested.

• Recommend more comparisons to yeast expression of paralogous RP.

As abovementioned, we modified the text in the introduction and discussion to highlight comparison with yeast RPs/duplicated genes.
