# Supplementary material for: Leishmania Ribosomal Protein (RP) paralogous genes compensate each other’s expression maintaining protein native levels
Source: PLoS One. 2024 May 16;19(5):e0292152. doi: 10.1371/journal.pone.0292152 (PMC11098316; doi:10.1371/journal.pone.0292152)
Supplement: Attachment — Submitted filename: Critique of PONE-D-23-29797_R1.docx [file pone.0292152.s012.docx]

Critique of PONE-D-23-29797_R1.

The authors have addressed some of my issues, but the most serious criticism (Figure 5) stands. I cannot recommend publication until this is resolved.

1. Thank you for referencing the universal nomenclature. This facilitates the comparison to articles about other species.
2. I appreciate that the authors inserted scales for the A254 profiles of the sucrose gradients (Figure 5). However, my comment was not directed towards the comparison of strains, but rather the comparison of the profiles for M199 grown cultures (blue trace) with starved culture (red trace). The former peaks go off the scale and the patterns cannot be compared to the latter. Since the Materials and Methods indicate that 5x10^8^ cells were harvested from both growing and starved cultures the very significant differences in signal intensity of the sucrose gradient A254 traces suggest that much more A254 material from the M199 cultures was loaded on the gradients as compared to the starved cultures. That is, the yield of A254 material was much greater from the M199 cultures than from the starved cultures. Why? Is the ribosome concentration decimated during starvation? A certain loss of ribosome density is expected but the extent indicated by the comparison of the blue and red traces is surprising. Is the lysis of the starved cultures inefficient? This issue should be further investigated by
   a. tabulating the A254 yield of the cleared lysates and by quantitative northern analysis of rRNA in the lysates
   b. rerunning the gradients with smaller amounts of material from the M199 cultures. It is essential to see how that trace changes during the starvation procedure. The pattern of the 40S-80S region does not look normal, even for the parental strain. Furthermore, the comparisons of peak heights in the revised manuscript is not understandable at all
   c. running quantitative northern analysis on samples of all sucrose gradient fractions in order to identify peaks of the individual rRNA species.
3. The authors fail to address the issue of the peak running slightly ahead of the 80S. The peak does certainly not show the expected sedimentation rate of a disome peak (said on the basis of evaluating theoudsans of sucrose gradients over the years). I agree with the authors that the separation of the 80S and named peak is not complete, but running northern analysis of samples from each fraction would show if there is an rRNA peak that co-sediments with the named peak. If the authors are correct about a peak of L13 with mRNA, this should not be the case. The authors spend a lot of the text discussing potential extra-ribosomal functions of ribosomal proteins. Thus, it is essential to settle whether the experiments presented support this idea or not.

Other issues

1. Explain abbreviations such as E-64, MTT, TKM, and others throughout.
2. Line 44: human ribosomes have 47 60S ribosomal proteins.
3. Line 253: buffer lysis > lysis buffer.
4. Line 254: something missing after the end of the parenthesis.
5. Line 254: agitation in a pipette; add the type of pipette and how many times, which could affect lysis efficiency.
6. Figure 1: Define asterisks.
7. Line 298: are the insertions also confirmed by sequencing?
8. Quantify blots throughout.
9. Indicate the base value of the log base in Figure 3B.
10. Line 395: I suggest that the text be changed to “which were completely reversed”
11. Figure 4D and Figure 6: What are the units on the histogram scale?
12. Line 431: The 60S peak is already reduced relative to the 40S peak in the pT007 culture.
13. Line 458 and in Figure 5: Free polysome (FP)> polysome free (PF).
14. Figure 5C: Puromicyn> Puromycin.
15. Line 516: not up-to-date; see e.g. papers from Maria Barnes’ group
16. Line 572: The authors should mention the effect of extra-ribosomal proteins on o53 stability.
