# Supplementary material for: Leishmania Ribosomal Protein (RP) paralogous genes compensate each other’s expression maintaining protein native levels
Source: PLoS One. 2024 May 16;19(5):e0292152. doi: 10.1371/journal.pone.0292152 (PMC11098316; doi:10.1371/journal.pone.0292152)
Supplement: Attachment — Submitted filename: Critique of third submission.docx [file pone.0292152.s013.docx]

Critique of the third submission

The authors have responded to my critique of Figure 5 by eliminating the results of the starvation experiment. I applaud his decision since it will take some time to complete this investigation and the starvation experiment is not essential for interpreting the expression of the L16a and L13 decision alleles. See however below.

The authors also addressed many of my comments under “Minor Points” but a few points need to be addressed.

1. Were the myc insertions sequences?
2. Some blots, e.g. Figure 2c, should be quantified.
3. The estimates of the peak heights in Figure 5 are not done correctly. The baselines for the 40S and 80S peaks is not at the bottom of the “valleys” between peaks, because the different peaks overlap. The best way to compare is to overlay the A254 tracings, line up the corresponding peaks, and graphically adjust the 40S peaks to have equal height. The authors arrive at the correct conclusion, but the procedure in not correct. Here is an example:

4. Line 264-265: Absolute OD is not a proper term, since OD, most correctly called A (absorption), is defined as the log_10_ of the ratio between the light transmission in a control solution relative to the experimental solution.
