# Supplementary material for: Leishmania Ribosomal Protein (RP) paralogous genes compensate each other’s expression maintaining protein native levels
Source: PLoS One. 2024 May 16;19(5):e0292152. doi: 10.1371/journal.pone.0292152 (PMC11098316; doi:10.1371/journal.pone.0292152)
Supplement: Attachment — Submitted filename: 240115_Response_2024_FINAL.docx [file pone.0292152.s015.docx]

**Critique of PONE-D-23-29797_R3.**

**Reviewer #2:** Thank you for your responses to R2. However, I see two outstanding issues:
1. Please define the calculation and scale for "band skew”. I brought this is in my comments on version R1.

We apologize for the laconic explanation given on R1. The band intensity on western blots was determined by ImageJ using “ROI manager” (ROI = Regions of Interest); the tool allows quantification of all bands, based on their size and intensity. Inside the ImageJ software, the array can be segmented into ROIs of identical size, where each ROI corresponds to one protein, certifying that the same area was considered for future comparisons between the samples. Thus, band skew shows how much the band intensity decreased comparing the RP expression in the presence or absence of its paralogue (*Nat Protoc*. 2016 August; 11(8): 1508–1530. doi:10.1038/nprot.2016.089).

2. The myc insertions have not been sequenced. I think this should be done.

We will ask reviewer #2 to reconsider this specific demand for the reasons below. We agree that sequencing knockouts, tagging or any genome editing is the most robust form of confirming the expected modification, without any further undesirable event, or off-target insertion. Nevertheless, I want to raise some points that might convince Reviewer #2 that, in this case specifically, we have robust evidence that both knockout and *myc* tags occurred as aimed. First aspect to consider is that genome editing in *Leishmania* has been first achieved in 1990, using the classical method of gene replacement that had been previously employed for *Saccharomyces cerevisiae*. We (Cruz and Beverley, 1990) pioneered the field of *Leishmania* genetic manipulation when we showed that “Following introduction of a construct containing dihydrofolate reductase–thymidylate synthase (*dhfr-ts*) flanking sequences fused to neomycin phosphotransferase, 45% of the colonies contained the planned homologous replacement; this frequency rose to nearly 100% in transfections using low amounts of DNA. Integrative transfection in *Leishmania* thus resembles that of *Saccharomyces cerevisae* in giving predominantly homologous events.” (<http://dx.doi.org/10.1038/348171a0>). This pioneer work demonstrated that the parasite has a highly efficient machinery of homologous recombination, which largely differs from most eukaryotes; insertion does not occur randomly and even when we exceeded in the amount of DNA to be transfected, what we observed was multiple insertions of the fragment in the homologous sequence/aimed site (same article above). The establishment of the CRISPR-Cas9 in these parasites (doi: 10.1098/rsos.170095) came to facilitate the previously labor-intensive and time-consuming approach, and represents a significant advance in the field. Even after 6 years of CRISPR/Cas9 use in *Leishmania*, we find no publications reporting off-target editing in *Leishmania*, and there are authors that report complete sequencing of the genome of transfected parasite, with no off-target events, this is probably partially due to the intrinsic genetic features of the organism. In addition to the abovementioned, we must consider that the results we report now compose a robust indication that *myc* insertion occurred in the expected genomic site.

The tag insertion in the RP genes studied in our work was also confirmed based on immune detection, as we used S16 antibody; the subcellular distribution of the tagged proteins using α-*myc* antibody was undistinguishable from the distribution of RPS16 protein detected by a specific α-S16 antibody (see supplementary material SF2). We have also used S16 polyclonal antibody in WB and the pattern was the same (not shown). The compensatory effect that maintains protein levels at similar levels comparing KO with wild type parasites was coherent, for all the paralogues (S16 and L13), showing that we are dealing with the protein we aimed to tag. For these reasons, we believe that the conventional PCR was an adequate tool in this study and confirmed the high efficiency of homologous recombination in *Leishmania* parasites, for both knockout and tagged parasites generation.
