# Supplementary material for: Leishmania Ribosomal Protein (RP) paralogous genes compensate each other’s expression maintaining protein native levels
Source: PLoS One. 2024 May 16;19(5):e0292152. doi: 10.1371/journal.pone.0292152 (PMC11098316; doi:10.1371/journal.pone.0292152)
Supplement: Attachment — Submitted filename: 240331_Response_Reviewer.docx [file pone.0292152.s016.docx]

**Critique of PONE-D-23-29797_R4.**

**Reviewer #2:** I know the standard procedure. However, "Band Skew" is, to my knowledge, not a standard term (I did not know it and I have read a lot of papers and worked in the field for a long time). As such Band Skew should be defined in Materials and Methods. And the scale should be defined (log2 or log10 change of expression). Note that I twice specifically requested that the scale be defined.

**Author’s comment:** In image processing, skewness (or band skew) refers to a measure of the asymmetry of the pixel intensity distribution within an image (doi:10.1167/10.9.3*)*. This parameter helps to quantify the degree to which the pixel values deviate from a symmetrical distribution around the mean. Thus, positive skewed distribution means that the right tail of the intensity values is longer or fatter than the left tail, indicating that there are more high-intensity pixels (doi:10.1167/10.9.3*)*. So, in our case, we used the ROI manager toll from ImageJ software to determine the intensity of the bands, being directly correlated to the protein amount. Considering this, our comparisons present a positive increment in the band skew, showing an increment in the pixel’s intensity/protein amount. Additionally, skewness itself does not have a specific unit in the context of image processing. It is a statistical measure used to describe the shape of the pixel intensity distribution, providing information about the asymmetry of the distribution without being tied to a particular unit (<https://brownmath.com/stat/shape.htm>). We added such specification about skewness/band skew in the Methods section.

**Reviewer #2**: I am fully convinced of the authors’ experience in manipulating the Leishmania genome. However, my concern is not whether insertions and tags were successfully placed in the S16 genes. Rather, in spite of the efficiency of CRISPR, it seems possible that the target sequence is not copied accurately into the genome, which could lead to a change of the amino acid sequence of the tag, and thus a change in the antibody affinity for the tag. This should not affect the relative expression of a specific paralog but may affect the estimate of the relative expression of the two paralogs. Can the authors conclusively refute this scenario?

**Author’s comment:** All tags were included at the 5’-end of the genes. Specific primers were used to amplify the tagged region, from the tag (*myc*) insertion to the CDS of each gene. Amplicons were cloned into pCR4-TOPO plasmid and sequenced by the Sanger method using M13 primers. The results show that all RP targeted genes investigated were successfully tagged using 3x*myc* and the three copies of the tag show no changes of the amino acid sequence. The sequencing results confirm that the tag insertion occurred as planned at the targeted locus with accuracy and in-frame. Sequencing results were added as supplementary material (SF6).

SF6. Sequencing results for all tag insertions.

,
